# Young adults’ familiarity with different cannabis product terms: the need for standardized cannabis surveys

**DOI:** 10.1186/s42238-022-00125-0

**Published:** 2022-04-01

**Authors:** S. Berberian, M. L. Broussard, C. Tully, V. Methuku, D. A. Pardini, M. H. Meier

**Affiliations:** 1 Department of Psychology, Arizona State University, PO Box 871104, Tempe, AZ 85287-1104 USA; 2 College of Health Solutions, Arizona State University, Phoenix, AZ USA; 3 Department of Criminology and Criminal Justice, Arizona State University, Phoenix, AZ USA

## Abstract

**Background:**

Cannabis legalization has resulted in the proliferation of cannabis products. Participants’ familiarity with terms for these products may have implications for assessment, as unfamiliarity with particular terms may result in under-reports of use.

**Methods:**

A convenience sample of 861 college students from one U.S. university completed a survey in the spring of 2020 about their familiarity with a variety of cannabis product terms and use of a variety of cannabis products.

**Results:**

Participants varied in their familiarity with cannabis product terms. For example, with regard to terms for cannabis concentrates with very high concentrations of THC, 85% of participants reported being familiar with the term “wax pen or THC oil,” but only 27% reported being familiar with the term “butane hash oil (BHO)” (i.e., the oil that composes most concentrates). Moreover, of participants who reported use of concentrates based on selecting pictures of the products they had used (*n* = 324, 40%), 99% (*n* = 322) reported having seen a “wax pen or THC oil” based on a written list of product terms, whereas only 20% (*n* = 65) reported having seen “butane hash oil (BHO).” This suggests that asking about use of “butane hash oil” use may result in lower rates of cannabis concentrate use than asking about use of “wax pen/THC oil.” With regard to terms for marijuana flower, 29% of participants (*n* = 248) reported being unfamiliar with the term marijuana “buds or flowers.” Of participants who reported use of marijuana flower based on selecting pictures of the products they had used (38% of the sample, *n* = 329), only 86% (*n* = 282) reported having seen marijuana “buds or flowers” based on a written list of product terms. This suggests that asking about use of marijuana “buds or flowers” use could result in under-reporting due to lack of familiarity with that term. Finally, when asked to select pictures of the cannabis product(s) that participants thought constituted “marijuana,” participants most commonly selected pictures of marijuana flower (93%), followed by wax pen/THC oil (57%) and edibles (49%).

**Conclusions:**

Young adults vary in their familiarity with cannabis product terms, and some may under-report cannabis use in surveys that rely on written cannabis product terms. Young adults also differ in terms of which cannabis products they think constitute “marijuana.” Although participants’ familiarity with specific cannabis product terms in this sample may not generalize to other populations, results highlight the need for standardized surveys of cannabis use that incorporate pictures of cannabis products to overcome issues related to variability in familiarity with cannabis product terms.

**Supplementary Information:**

The online version contains supplementary material available at 10.1186/s42238-022-00125-0.

## Introduction

Cannabis legalization in the USA has resulted in the rapid growth of the cannabis industry and the proliferation of cannabis products (e.g., marijuana flower, various forms of concentrates, and edibles). The risks associated with use of these products might vary due to differences in THC content and method of administration. For example, the THC concentration of marijuana flower sold in dispensaries is approximately 20% (Cash et al. 2020; Smart et al. 2017), whereas the THC concentration of cannabis concentrates can exceed 80-90% (Chandra et al. 2019; NIDA 2020; Smart et al. 2017). Higher THC concentrations could have negative implications for health (Wilson et al. 2019), such as increased risk for cannabis use disorder (Chan et al. 2017; Curran et al. 2019; Meier, 2017), psychosis (Di Forti et al. 2015; Moore et al. 2007), and use of other illicit drugs or alcohol (Fedorova et al. 2019; Frohe et al. 2018). Different methods of cannabis administration also have different health risks. Smoking by combustion (e.g., joint or pipe) involves inhalation of carcinogens (Meehan-Atrash et al. 2019); vaping can expose users to residual solvents in concentrates (Raber et al. 2015), with some black-market THC vapes linked to severe lung injury (Abeles et al. 2020); and consumption of cannabis edibles, due to their slow absorption and risk for overdose, can result in cannabis intoxication-related emergency room visits (Bui et al. 2015). Therefore, it is crucial to accurately assess which cannabis products and administration methods people use, as the associated risks might differ.

Scientists and cannabis users may use different terms to refer to the different cannabis products. Until recently, the term “marijuana” had the same meaning for scientists and cannabis users, because marijuana flower was the main cannabis preparation available. Now, researchers use the terms “buds” or “flower” to differentiate herbal marijuana from other cannabis preparations, such as concentrates and edibles (Bidwell et al. 2020; D'Amico et al. 2020; Fedorova et al. 2019; Kilmer et al. 2013; Kruger and Kruger 2019). For example, two recent studies asked, “On a typical use day, how much marijuana flower/bud do you personally consume?” (D'Amico et al. 2020; Kilmer et al. 2013). Yet, it is unknown if “buds” and “flower” are terms that are familiar to cannabis users. Additionally, recent research has assessed cannabis concentrate use by asking about use of “dabs,” “butane hash oil” (BHO),” or “concentrates” in self-report measures (Barrington-Trimis et al. 2020; Stogner and Miller 2015; Meier 2017; Meier et al. 2019), but it is not clear which terms for concentrates are familiar to cannabis users. Moreover, some terms (e.g., the term “dab”) refer both to a cannabis product and to an administration method. More clarity on the cannabis product terms that people use is needed. If cannabis users use different terms, and/or interpret cannabis terms used on surveys differently than each other and researchers, new and standardized measures of cannabis use are needed.

In an effort to understand young adults’ familiarity with cannabis product terms, we asked college students to report on whether they had ever heard of, or seen, various cannabis products using a written list of terms used colloquially, by industry, and by researchers in surveys of cannabis use. Next, we asked participants to report on whether they had used different cannabis products based on product pictures, and then we determined whether those who had used a particular product were also familiar with the corresponding written cannabis product terms. Finally, participants were asked to select pictures of the cannabis products they considered to be “marijuana” and to select the best-fit terms for pictures of different cannabis products.

## Methods

### Participants and procedure

Participants were college students enrolled in introductory psychology courses at a large southwestern university in the USA in the spring of 2020. At that time, medical cannabis use was legal but recreational use was not. Our 30-item questionnaire was embedded within a larger screening survey administered to the participant pool. Due to a large number of investigators using the pool, our survey was assigned to a subset of participants—i.e., those born in even-numbered months (*N* = 861). Participation was voluntary and anonymous. All participants provided informed consent. Participants were notified if they skipped a survey question and were given the opportunity to answer the question, which resulted in no missing data. All participants who completed the survey received course credit. This study was approved by the university’s institutional review board. This study follows STROBE guidelines (Additional file 1). Participants’ demographics are shown in Table 1.

**Table 1:** Participant demographics (*N* = 861 college students)

Participant demographics	*N*	%/M (SD)
Age	861	19 (2.09)
Sex (% male)	365	42.4
Race		
White/Caucasian	511	59.3
Hispanic/Latino	195	22.7
Asian/Pacific Islander	173	20.1
Black/African American	55	6.4
Middle Eastern	29	3.4
Native American	27	3.1
Socioeconomic status		
Upper class	45	5.3
Upper-middle class	289	33.8
Middle class	336	39.2
Lower-middle class	116	13.6
Working class	67	7.8
Parental Education		
Graduate/professional	81	9.5
Master’s	187	22.0
4-year college	302	35.5
Some college or 2 years	124	14.6
High school diploma/GED	44	5.2

### Measures

#### Cannabis familiarity

First, participants were asked about their familiarity with a variety of cannabis product terms using a written list of terms generated from complementary sources: (1) the cannabis dictionary on Weedmaps (https://weedmaps.com/learn/dictionary), (2) published research articles, and (3) interviews conducted with young people. Participants were given the following directions: “There are now several forms of marijuana that are referred to by a number of different names. Please indicate whether you have ever heard of or seen (in real life or in pictures) the following forms of marijuana.” Participants were then presented with the following product terms: “buds or flowers,” “wax pen or THC oil,” “shatter,” “rosin,” “budder or badder,” “crumble,” “hash,” “diamonds, THCa crystals, or crystalline,” and “butane hash oil (BHO), CO2 oil, Rick Simpson oil, or honey oil.” Response options were 1 (never heard of it or seen it), 2 (heard of it but haven’t seen it in real life or in pictures), or 3 (have seen it in real life or in pictures). Questions were not accompanied by pictures.

Next, participants were shown pictures of the different cannabis products (i.e., marijuana flower, wax pen/THC oil, shatter, rosin, budder/badder, crumble, hash, diamonds]), and were asked to select the term (same terms as above) that best fit what they thought most people would call that cannabis product.

#### Participants’ definition of “marijuana”

Next, participants were shown pictures of the different cannabis products and were asked to “select the picture or pictures that represent what you mean when you use the term ‘marijuana.’”

#### Cannabis use

At the end of the survey**,** participants were asked whether they had used various cannabis products using example pictures of these products. Separate pictures were shown for marijuana flower, wax pen or THC oil (i.e., a group of pictures depicting cartridges of liquid cannabis concentrates that are vaped), shatter, rosin, budder/badder, crumble, hash, and THCa crystals/diamonds. Pictures are available from the corresponding author. If a participant reported lifetime use of a specific cannabis product, they were asked about their frequency of use in the past year using response options ranging from 1 (did not use in the past year) to 6 (daily use). As with the lifetime use questions, all past-year frequency questions were asked using pictures.

### Statistical analysis

We computed the percentage of participants who reported being familiar with the various written cannabis product terms for the full sample and for the subset of participants who reported lifetime and past-year use of the corresponding product based on selecting pictures of the products they had used. We also computed the percentage of participants who selected particular cannabis product terms as the “best fit” term for each product picture, and the percentage of participants who included particular cannabis product pictures in their definition of “marijuana.” Finally, we estimated the correlation between the number of cannabis products ever used and the number of cannabis products a participant considered to be “marijuana,” as a means of quantifying the extent to which more experienced users had broader definitions of marijuana than less experienced users.

## Results

Table 2 shows the percentage of participants who reported being familiar or unfamiliar with various cannabis product terms. Percentages are shown for the full sample and for the participants who reported lifetime and past-year use of the corresponding product, with use based on selecting pictures of the cannabis products they had used. In the full sample, the most familiar term was “wax pen or THC oil,” with 85.0% of participants reporting they had heard of (13.8%) or had seen (71.2%) this cannabis product. By comparison, only 71.2% of the full sample was familiar with the term marijuana “buds or flowers.” Moreover, of participants who had used a wax pen/THC oil (based on selecting pictures of the products they had used, *n* = 324), 99.4% (*n* = 322) reported having seen a “wax pen or THC oil.” By comparison, only, 85.7% of participants who had used marijuana flower (*n* = 282/329) reported having seen it when initially asked about whether they had heard of or seen marijuana “buds or flowers.”

**Table 2:** Cannabis product familiarity in the full sample (*N* = 861 college students) and in subsamples of lifetime and past-year users of each specific type of cannabis

	Full sample						Lifetime users of the cannabis type						Past-year users of the cannabis type					
Familiarity	Never		Heard		Seen		Never		Heard		Seen		Never		Heard		Seen	
Cannabis term	%	*n*	%	*n*	%	*n*	%	*n*	%	*n*	%	*n*	%	*n*	%	*n*	%	*n*
Buds/flowers	**28.8**	248	**16.5**	142	**54.7**	471	**6.1**	20	**8.2**	27	**85.7**	282	**6.2**	16	**7.3**	19	**86.5**	224
Wax pen^a^	**15.0**	129	**13.8**	119	**71.2**	613	**0.0**	0	**0.6**	2	**99.4**	322	**0.0**	0	**0.4**	1	**99.6**	265
Shatter	**75.8**	652	**8.0**	69	**16.2**	139	**12.9**	11	**2.4**	2	**84.7**	72	**8.3**	5	**1.7**	1	**90.0**	54
Rosin	**73.4**	632	**14.9**	128	**11.7**	101	**23.2**	16	**14.5**	10	**62.3**	43	**19.2**	9	**12.8**	6	**68.1**	32
Budder^b^	**81.6**	702	**9.0**	77	**9.4**	81	**7.5**	3	**10.0**	4	**82.5**	33	**0.0**	0	**4.0**	1	**96.0**	24
Crumble	**77.2**	665	**10.6**	91	**12.2**	105	**0.0**	0	**0.0**	0	**100.0**	35	**0.0**	0	**0.0**	0	**100.0**	22
Hash	**44.8**	386	**32.6**	281	**22.5**	194	**0.0**	0	**0.4**	1	**96.6**	28	**0.0**	0	**0.0**	0	**100.0**	17
Diamonds^c^	**57.0**	491	**27.6**	238	**15.3**	132	**3.7**	1	**0.0**	0	**96.3**	26	**0.0**	0	**0.0**	0	**100.0**	16
BHO^d^	**72.9**	628	**17.0**	146	**10.1**	87	**60.8**	197	**19.1**	62	**20.1**	65	**59.4**	158	**20.3**	54	**20.3**	54

Note. This table reports on the percentage of participants who had never heard of, heard of but not seen, or seen various cannabis products based on a written list of product terms. *Never* = never heard of. *Heard* = heard of but not seen. *Seen* = heard of and seen. *Lifetime Users* = the subsample of participants who reported lifetime use of each cannabis product type based on a picture of that product. For example, of the 329 people who reported lifetime marijuana buds/flowers use based on a picture of buds/flowers, 6.1% reported never having heard of “buds or flowers,” 8.2% reported having heard of it but had not seen it, and 85.7% reported having seen it. *Past-Year Users* = the subsample of participants who reported past-year use of each product based on a picture of that product ^a^Wax pen specified “Wax pen/THC oil”

^b^Budder specified “Budder or badder”

^c^Diamonds specified “Diamonds, THCa crystals, or crystalline”

^d^BHO specified “Butane hash oil (BHO), CO2 oil, Rick Simpson oil, or honey oil.” Percentages are bolded to facilitate comparison across terms and groups

Table 3 shows the best-fit terms for pictures of the different cannabis products. For pictures of marijuana flower and wax pen/THC oil, most participants selected the terms “buds or flowers” (69.0%) and “wax pen/THC oil” (87.0%) as the best-fit terms, respectively. However, for other cannabis products (shatter, rosin, budder, crumble, hash, diamonds), no term was selected by a majority of participants. Strikingly, 87.0% of participants selected “wax pen or THC oil” as the best-fit term for vaped concentrates, and only 1.7% selected BHO as the best-fit term.

**Table 3:** Cannabis term chosen as “best-fit term” when shown a picture of the product (*n* = 861 college students)

Picture shown	Marijuana flower		Wax pen/THC oil		Shatter		Rosin		Budder		Crumble		Hash		Diamonds	
Term chosen	%	*n*	%	*n*	%	*n*	%	*n*	%	*n*	%	*n*	%	*n*	%	*n*
Buds/flowers	**69.0**	594	0.1	1	.1	1	.1	1	0.2	2	1.2	10	1.4	12	0.1	1
Wax pen^a^	0.2	2	**87.0**	749	3.3	28	12.3	106	1.9	16	0.6	5	0.2	2	0.4	3
Edibles	0.7	6	0.0	0	0.1	1	0.2	2	1.1	9	.01	1	1.9	16	0.5	4
Shatter	0.0	0	0.0	0	**44.0**	379	5.9	51	0.7	6	2.7	23	2.6	22	2.4	21
Rosin	0.2	2	0.1	1	2.8	24	13.8	119	4.9	42	4.0	34	2.4	21	4.0	34
Budder^b^	1.3	11	0.1	1	0.7	6	6.0	52	24.7	212	5.3	46	1.6	14	1.6	14
Crumble	2.8	24	0.0	0	1.2	10	0.5	4	1.1	9	26.6	229	16.7	146	3.0	26
Hash	5.1	44	0.1	1	0.5	4	1.6	14	2.3	20	4.0	34	14.6	126	0.2	2
Diamonds^c^	0.0	0	0.0	0	11.0	94	0.9	8	0.5	4	1.1	9	1.4	12	**43.4**	374
BHO^d^	0.2	2	1.7	15	3.7	32	16.6	143	8.8	76	1.2	10	1.2	10	2.3	20
Do not know	20.4	176	10.8	93	32.8	282	**41.9**	361	**54.0**	464	**53.4**	460	**55.8**	480	42.0	362

*Note:* The column headings represent the products shown in pictures, and the row headings represent the terms participants selected as the best-fit term for the picture. The most chosen response option is bolded

^a^Wax pen question specified “Wax pen/THC oil”

^b^Budder specified “Budder or badder”

^c^Diamonds question specified “Diamonds, THCa crystals, or crystalline"

^d^BHO question specified "Butane hash oil (BHO), CO2 oil, Rick Simpson oil, or honey oil”

**Fig. 1:**
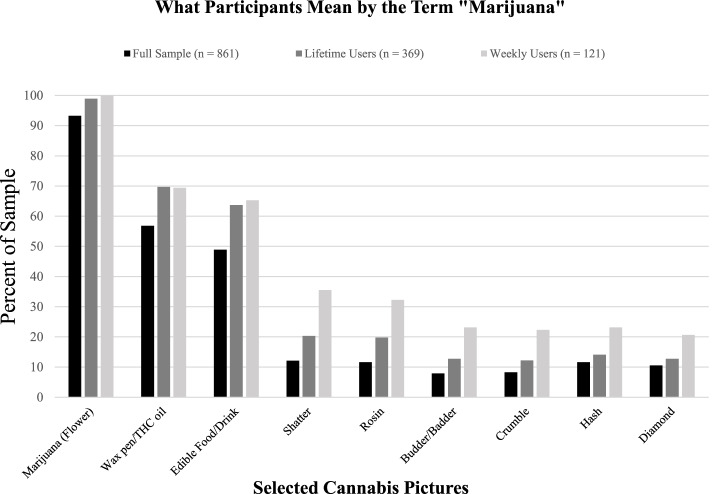
Percentage of participants (*N* = 861 college students) who selected each cannabis product picture as part of their definition of “marijuana”. Note: participants were asked to select picture(s) of cannabis products that they include in their definition of “marijuana.” This figure shows the percentage of the sample who selected each picture

Figure 1 shows the cannabis products that participants considered to be “marijuana.” The percentage of participants who selected a particular cannabis product picture as “marijuana” is shown for the full sample, lifetime cannabis users (i.e., participants who used any cannabis product in their lifetime), and past-year weekly cannabis users (i.e., participants who reported using any cannabis product at least once a week in the past year). The picture of marijuana flower was commonly selected to mean “marijuana” (93.3% in the full sample), whereas pictures of wax pens/THC oil and edibles were less commonly selected (56.8% and 48.9% in the full sample, respectively). Results were similar in the subsample of lifetime and weekly cannabis users, except users generally selected a larger number of cannabis products as “marijuana” than nonusers. On average, participants selected *M* = 2.62 (SD = 2.05) of nine different cannabis products as “marijuana.” A moderate correlation (*r* = 0.37, *p* = < .001) was found between the number of cannabis products ever used and the number of cannabis products a person considered to be “marijuana,” suggesting that more experienced users have broader definitions of “marijuana” than less experienced users.

## Discussion

With cannabis legalization and commercialization in the USA, the number of cannabis products has greatly proliferated. There is an urgent need for standardized, reliable, and valid assessments of use of these products. Our study highlights this need by showing three key findings. First, young adults varied in their familiarity with cannabis product terms. Second, a subset of young adults who reported using a cannabis product based on a picture of that product also reported never having seen that product based on written product terms, suggesting that use of some cannabis product terms in surveys of cannabis use may result in under-reports of use. Third, young adults differed in terms of which cannabis products they included in their definition of “marijuana.”

With regard to young adults’ familiarity with cannabis product terms, the most familiar term for cannabis concentrates was “wax pen/THC oil.” Most young adults had at least heard of “wax pen/THC oil,” whereas far fewer had heard of “butane hash oil (BHO),” “crumble,” “shatter,” “budder,” and “rosin.” Concordantly, when shown a picture of a wax pen/THC oil, 87% of cannabis users and nonusers chose “wax pen or THC oil” as the best-fit term to identify the product, whereas only 1.7% chose “Butane hash oil (BHO), CO2 oil, Rick Simpson oil, or honey oil.” Unlike “wax pen or THC oil,” which was familiar to 85% of participants, only 71% of the sample had heard of the term marijuana “buds or flowers.” This is especially important when considering that the prevalence of marijuana flower use and wax pen/THC oil use in the sample was about the same, based on participants responses to pictures of the cannabis products they had used (38.2% and 37.6%, respectively). This suggests that studies that query use of marijuana “buds or flowers” might underestimate the prevalence of marijuana flower use, because some young adults who have used marijuana flower are unfamiliar with this term. In general, cannabis product terms were more familiar to young adults who had used these cannabis products, but even some users were unfamiliar with some terms, particularly rosin and BHO.

With regard to what young adults mean when they use the term “marijuana,” over 90% of young adults selected pictures of marijuana flower, and only about half of young adults selected pictures of wax pens/THC oil and edibles. More experienced users generally included more cannabis products in their definition of “marijuana.” This is important because the term “marijuana” or “marijuana or hashish” is used in many large epidemiological surveys, including Monitoring the Future and the National Survey on Drug Use and Health (Boyd et al. 2015; Hasin et al. 2015). The variation in individual meanings of the term “marijuana” recommends caution when using this term in survey research, or, at the very least, defining for participants that the term “marijuana” includes edibles and other non-flower forms of cannabis.

The results of this study must be considered in the context of limitations. First, our sample comprised a convenience sample of college students from a single state that had legalized medical but not adult recreational cannabis use. Participants were mostly white and from middle to upper-class families. Although our results might recommend using certain product terms over others (e.g., “wax pen/THC oil” as opposed to “BHO” to refer to vaped concentrates), and recommend caution regarding use of the term “marijuana,” there may be cultural and regional variation in cannabis product terminology that limits the generalizability of these findings. Nonetheless, college students show high rates of cannabis use, both here and in representative samples. For example, the 2020 Monitoring the Future survey of college students showed that the annual prevalence of “marijuana or hashish” use and marijuana “vaping” was 43.9% and 24.6%, respectively. Our study suggests that even in populations characterized by high rates of cannabis use, there is considerable unfamiliarity with particular cannabis product terms. This study calls attention to under-recognized issues associated with querying cannabis use using written cannabis product terms that likely extend beyond this particular sample. Second, our list of cannabis product terms, and our array of cannabis product pictures, were not comprehensive. Given cannabis commercialization in the USA, and the associated proliferation of cannabis products, generating a comprehensive list of cannabis products and terms is a challenge. Third, participants self-reported their cannabis use, and some evidence suggests that participants both under- and over-report use. Nonetheless, self-reports have been found to be fairly accurate (Loflin et al. 2020), and most epidemiological surveys rely on self-reports.

## Conclusions

Young adults vary in their familiarity with cannabis product terms, and these differences could impact the estimated prevalence of cannabis use, as well as our understanding of the predictors and consequences of use of particular cannabis products. The different cannabis products vary in terms of typical THC concentration, contamination by residual solvents and pesticides, and risks associated with the route of administration of the product, all which may be important determinants of health (Alzghari et al. 2017; Ashley et al. 2020; Raber et al. 2015). Therefore, standardized surveys of cannabis use are needed, and surveys that use pictures of the different cannabis products could potentially address some of the issues with terminology revealed here.

## Supplementary Information

**Additional file 1.** The STROBE checklist for cross-sectional study reporting guidelines.

## Data Availability

The datasets generated and analyzed during the current study can be made publicly available upon IRB approval and are available from the corresponding author upon reasonable request.

## Abbreviations

THC: Δ9-tetrahydrocannabinol
BHO: Butane Hash Oil
THCa: Δ9-tetrahydrocannabinolic acid
STROBE: Strengthening the reporting of observational studies in epidemiology
CO2: Carbon dioxide
